# The promising potential of piperlongumine as an emerging therapeutics for cancer

**DOI:** 10.37349/etat.2021.00049

**Published:** 2021-08-30

**Authors:** Dey Parama, Varsha Rana, Sosmitha Girisa, Elika Verma, Uzini Devi Daimary, Krishan Kumar Thakur, Aviral Kumar, Ajaikumar B. Kunnumakkara

**Affiliations:** Cancer Biology Laboratory and DBT-AIST International Center for Translational and Environmental Research (DAICENTER), Department of Biosciences and Bioengineering, Indian Institute of Technology Guwahati, Assam 781039, India; Changchun Institute of Applied Chemistry, Chinese Academy of Sciences, China

**Keywords:** Piperlongumine, piplartine, *Piper longum* Linn., cancer, signaling pathways, chemotherapy, radiotherapy, toxicity

## Abstract

In spite of the immense advancement in the diagnostic and treatment modalities, cancer continues to be one of the leading causes of mortality across the globe, responsible for the death of around 10 million patients every year. The foremost challenges faced in the treatment of this disease are chemoresistance, adverse effects of the drugs, and the high cost of treatment. Though scientific studies over the past few decades have foreseen and are focusing on the cancer-preventive and therapeutic potential of natural products and their underlying mechanism of action, many more of these agents are not still explored. Piperlongumine (PL), or piplartine, is one such alkaloid isolated from *Piper longum* Linn., which is shown to be safe and has significant potential in the prevention and therapy of cancer. Numerous shreds of evidence have established the ability of this alkaloid and its analogs and nanoformulations in modulating various complex molecular pathways such as phosphatidylinositol-3-kinase/protein kinase B/mammalian target of rapamycin, nuclear factor-kappa B, Janus kinases/signal transducer and activator of transcription 3, etc. and inhibit different hallmarks of cancer such as cell survival, proliferation, invasion, angiogenesis, epithelial-mesenchymal-transition, metastases, etc. In addition, PL was also shown to inhibit radioresistance and chemoresistance and sensitize the cancer cells to the standard chemotherapeutic agents. Therefore, this compound has high potential as a drug candidate for the prevention and treatment of different cancers. The current review briefly reiterates the anti-cancer properties of PL against different types of cancer, which permits further investigation by conducting clinical studies.

## Introduction

Regardless of the notable progress achieved in cancer diagnosis and treatment, it is still considered as one of the most dreadful and prevalent diseases having very high morbidity and mortality rate [1–10]. There are diverse types of cancer, all of which are associated with atypical growth and proliferation of cells leading to approximately 10 million deaths per year [11–16]. The majority of cancers occur due to genetic mutations associated with lifestyle and environmental-related factors, although some of the cancer types are caused because of inherited genetic makeup [10, 17]. A considerable proportion of the global cancer burden can be relieved by evading the risk factors, such as consumption of carcinogenic products, poor diet, and absence of physical activity leading to obesity, sexually transmitted diseases, and pollution, to name a few [17]. A diverse range of drugs have been discovered and screened with the aim to cure this disease in the last few decades. However, most of the standard chemotherapeutics fail to provide complete relief to the patients and are further known for imparting innumerable side effects and secondary diseases like myelosuppression, nausea, vomiting, heart diseases, hepatic dysfunction, hypertension, malaise, etc. In addition, extensive use of those drugs also results in the development of chemoresistance in cancer cells [18–22]. Therefore, advances in the development of novel, non-toxic, effectual, and cost-effective therapeutic modalities are an urgent requirement for the management of this life-threatening disease.

Since ancient times, plant-derived phytochemicals and herbal medicines are being explored as a treatment modality against different chronic diseases, including cancer, and thus have gained extensive popularity for possessing beneficial healing properties [23–35]. The healing properties of the plant-derived products are due to the presence of a wide range of biologically active alkaloids, flavonoids, tannins, diterpenoids, carotenoids, and phenolic compounds present in various parts [36–42]. Therefore, these compounds play a vital role in preventing cancer initiation and suppressing the process of cell proliferation, invasion, metastasis, and chemoresistance [43–52]. Interestingly, it was reported that, almost one-third of all the emerging drugs approved by the United States Food and Drug Administration (US FDA) consist of natural products either in their native form or their analogs [19, 53, 54]. Even though experts across the globe pay more attention to exploring the anti-inflammatory, anti-oxidant, anti-cancer, chemosensitizing, and radiosensitizing potential of the active compounds extracted from natural sources, several other phytochemicals are still left to be recognized [41, 53, 55–59].

**Figure 1.**
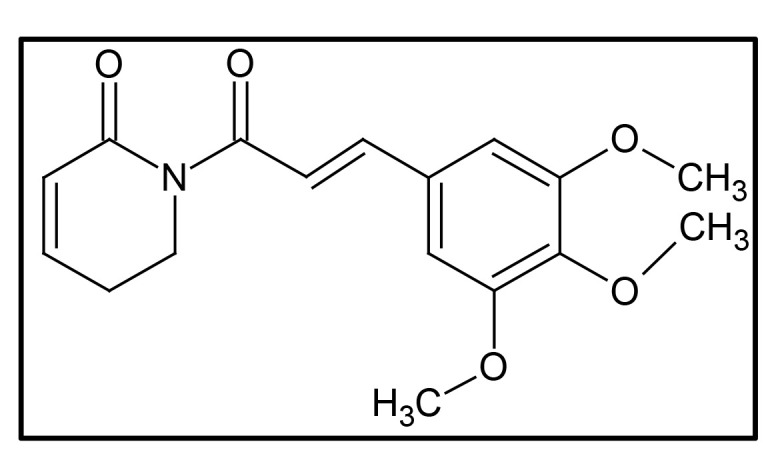
Chemical structure of PL

Piperlongumine (5,6-dihydro-1-[(2E)-1-oxo-3-(3,4,5-trimethoxyphenyl)-2-propenyl]-2(1H)-pyridinone, PL; Figure 1), also known as piplartine, is the major active alkaloid present in the fruits and roots of *Piper longum* Linn., commonly known as long pepper or “*Pippali*” belonging to the Piperaceae family [60–63]. Accumulating lines of evidence have documented its role in cancer prevention, and it is reported to be effective against many human cancer cell lines of breast, colon, liver, lung, prostate, skin, and thyroid. As PL is a naturally occurring alkaloid, it is comparatively less toxic, and it can also concomitantly modulate the expression of various target genes, thereby imparting potential anti-cancer effects [62].

## Sources of PL

**Figure 2.**
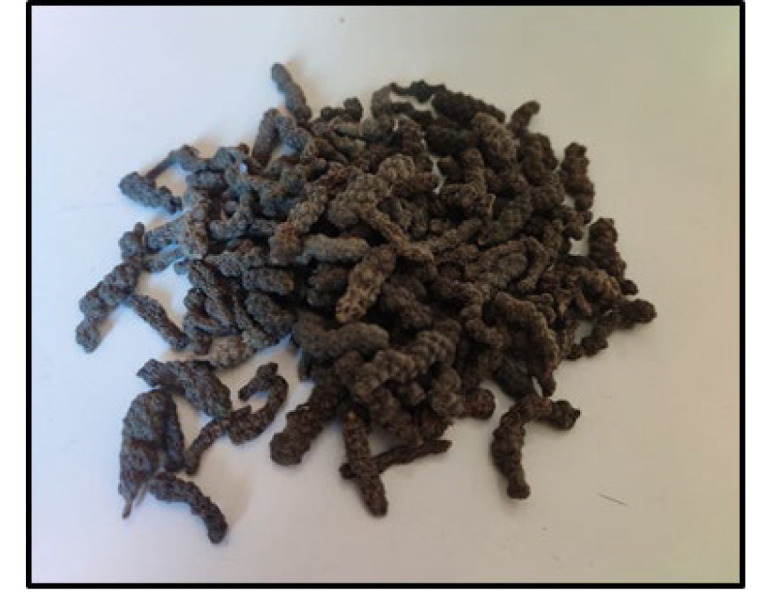
Source of PL

PL is extracted from the fruits and roots of *P. longum* L. (Figure 2), and was first isolated and characterized in 1963 [60, 64]. The plant grows in the wild tropical rain forests of India, Indonesia, Malaysia, Rhio, Timor, Nepal, Philippines, and Sri Lanka [65]. It has also been grown indigenously in India, along with cultivation in tropical and subtropical areas of Asian and Pacific islands [66].

## Biosynthesis and chemistry of PL

The chemical structure of PL is composed of two α,β-unsaturated imide functionalities [67]. The structure of PL was first determined in 1968, and generally, the linkage of 5,6-dihydropyridin-2(1H)-one to a 3,4,5-trimethoxyphenyl group via an E-acryloyl group form a molecule of PL [68]. Using the same components, He et al. [69] synthesize PL. The 5,6-dihydropyridin-2(1H)-one synthesized from commercially available 2-piperidone acted as one of the precursors. Another precursor, 3,4,5-trimethoxycinnamic acid, was converted to acyl chloride 3,4,5-trimethoxycinnamic acid using an oxalyl chloride. Then the sodium hydride deprotected 5,6-dihydropyridin-2(1H)-one was allowed to react with (*E*)-3-(3,4,5-trimethoxyphenyl) acryloyl chloride that gave rise to the resultant compound PL [69].

Similarly, Sun et al. [70] has synthesized PL by adding acyl chloride (or acid anhydride) to a 5,6-dihydropyridin-2(1H)-one and incubating the solution at 0°C followed by the addition of sodium hydride, and the solution was kept at 0°C for a couple of hours and then stirred for the next 18 h at room temperature in dry air. The solution was then transferred into ice water, and subsequent extraction was done with ethyl acetate. The organic phases were dried, concentrated, and purified to get the final product PL and its analogs through the flash column chromatographic method [70].

Another group has reported that the synthesis of PL involves the fusion of α,β-unsaturated δ-lactam with the cinnamates [71]. The *N*-Boc protection of the commercial compound, δ-valerolactam, using the di-tert-butyl dicarbonate leads to the synthesis of α,β-unsaturated δ-lactam with 98% yield. The *N*-Boc protected lactam compound derived from δ-valerolactam is converted to 3-(phenylthio) piperidin-2-one with the complementary treatment of freshly prepared lithium diisopropylamide and diphenyl disulfide [71]. The sulfide is then allowed to oxidize. The resultant sulfoxide is thermally eliminated, which gives rise to α,β-unsaturated δ-lactam, and it’s the *N*-Boc deprotection in an acidic environment that produced the desired lactam molecule. The phosphonoacetamide is then synthesized from lactam through the Arbuzov reaction. It is then allowed to mediate sodium hydride deprotonation and react with 3,4,5-trimethoxybenzaldehyde, giving rise to PL with 51% yield [71]. The 3,4,5-trimethoxybenzyl in PL is an anti-cancer exhibiting moiety that is employed in synthesizing the PL derivatives such as L50377 to improve anti-cancer efficacy [72].

PL is also combined with metal complexes such as ruthenium and platinum to increase its anti-cancer efficiency [73, 74]. In line with this, a novel PL and ruthenium complex was synthesized from two precursor complexes, such as [RuCl_2_(N-N) (P-P)] and 1,4-bis(diphenylphosphino)butane [73, 75]. Further, platinum-based PL derivatives were also synthesized using the demethylated PL, PIP-OH ligand that was allowed to react with the platinum precursor PtCl_2_(PPh_3_)_2_, and through the exchange of one chloride molecule, it resulted in a novel platinum-based PL complex, i.e., *cis*-[PtCl(PIP-OH)(PPh_3_)_2_]PF_6_ [74].

## Biological activities of PL

A plethora of studies have reported multiple biological properties of *P. longum* L., including anti-arthritic, anti-asthmatic, anti-diabetic, anti-epileptic, anti-inflammatory, anti-microbial, anti-oxidant, anti-stress, anti-tumor, anti-ulcer, as well as immunomodulatory properties [61, 66]. A study demonstrated the hepatoprotective effects of *P. longum* via decreasing the rate of lipid peroxidation and increasing glutathione (GSH) levels [76]. Furthermore, administration of the roots of *P. longum* displayed high antifertility effects [65]. Another study conducted by Khushbu et al. [77] has demonstrated the cardioprotective effect of *P. longum* against biochemical and histopathological damages in rat models suffering from acute myocardial infarction. Furthermore, the anti-thrombogenic properties of PL (50 mg/kg) were also observed in the pulmonary thrombosis mice model [78]. PL was also found to inhibit an essential rate-limiting enzyme, human aldose reductase, involved in the conversion of glucose to sorbitol. Diabetic complications are generally associated with sorbitol accumulation. Therefore, these insights point towards the anti-diabetic effects of PL [79].

Furthermore, a novel PL-mediated therapeutic strategy for atherosclerosis plaque revealed that PL acted via the suppression of platelet-derived growth factor (PDGF) receptor signaling [80]. An *in vivo* study demonstrated the anti-depressant and anxiolytic effects of PL through experimental analyses including open field, forced swimming tests, and elevated plus maze [81]. Interestingly, the synergistic effects of PL have also been observed with existing anti-bacterial drugs, including tetracycline and rifampicin, thereby improving their effectiveness [82]. Thus, the above reported biological properties reflect the vast therapeutic potential of PL in the treatment of various anomalies.

## Molecular targets of PL

**Figure 3.**
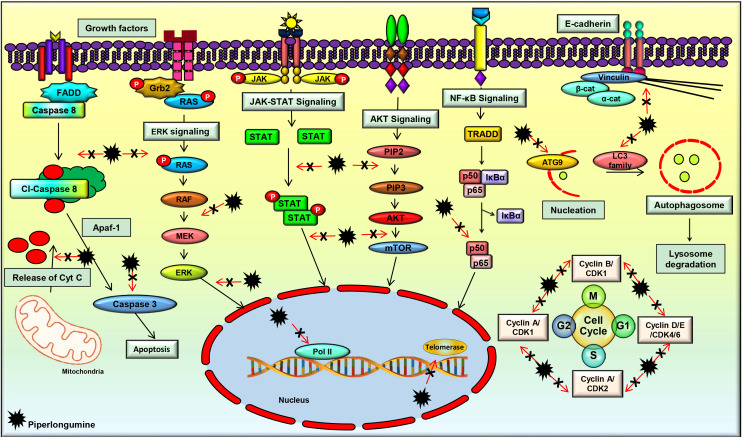
PL regulates the molecular targets and various signaling pathways involved in cancer progression

A plentiful of studies have evidenced the multi-targeted nature of PL, which contributes to its diverse pharmacological activities. It was found to modulate the important cell signaling pathways such as phosphatidylinositol-3-kinase (PI3K)/protein kinase B (Akt)/mammalian target of rapamycin (mTOR), nuclear factor-kappa B (NF-κB), Janus kinases (JAK)/signal transducer and activator of transcription 3 (STAT3), and extracellular signal-regulated kinase (ERK) which play a critical role in regulating the processes involved in the initiation, development, and progression of cancer. PL significantly downregulated the mRNA expression of the cell cycle regulatory genes such as *cyclin B1*, *cyclin D1*, cyclin-dependent kinases (*CDK*)-*1*, *CDK4*, *CDK6*, and proliferating cell nuclear antigen (*PCNA*) [83]. This compound also modulated the expression of cell survival and invasion associated genes such as heme oxygenase 1 (*HMOX1*), heat shock protein family A member 1A (*HSPA1A*), caspase-3 (*CASP3*), cyclin dependent kinase inhibitor 1A (*CDKN1A*), *MYC*, phosphatidylinositol-4,5-bisphosphate 3-kinase catalytic subunit gamma (*PIK3CG*), B-cell lymphoma 2 (*Bcl-2*), NF-κB subunit 1 (*NF-κB1*), *AKT3*, matrix metalloproteinase-9 (*MMP-9*), and *Twist* [84, 85] (Figure 3).

### PL and PI3K/Akt/mTOR signaling

The PI3K/Akt/mTOR pathway is an important cell signaling pathway that has a critical role in several cellular processes such as proliferation, growth, and cellular metabolism. The abnormality of this signaling pathway is a common cause of cancer, and many studies reported the downregulation of this pathway by PL [86–89]. For instance, PL was found to suppress this pathway in breast cancer cells, which led to cell apoptosis and autophagy [90]. In addition, inhibition of this pathway following PL treatment was observed in cervical cancer, colorectal cancer (CRC), and lung cancer cells as well, which resulted in the prevention of tumor growth [90–93]. Further, suppression of the Akt/mTOR pathway by PL was also associated with the partial inhibition of glycolysis, as suggested by a study on lung cancer cells [94]. Additionally, suppression of the Akt/mTOR signaling pathway by PL in renal cell carcinoma cells was demonstrated to be reactive oxygen species (ROS) dependent, which led to cell death and inhibition of tumor initiation and progression [90]. Furthermore, PL treatment suppressed lung tumor growth in an *in vivo* model by inhibiting the PI3K/Akt/mTOR pathway [95]. As this pathway regulates many other processes involved in cancer development and progression, further studies are warranted to delineate the effect of PL on these processes, which would pave the way in the management of different malignancies. In addition, novel analogs of PL and their interaction with this pathway can be explored, which would help to develop novel Akt/mTOR inhibitors for the treatment of different cancers.

### PL and NF-κB signaling

The NF-κB signaling pathway is known to play an active role in cell survival and proliferation. It regulates numerous physiological processes such as development, differentiation, inflammation, immunity, and metabolism in the initial and later stages of cancer [96–99]. Therefore, the agents that suppress the activation of this pathway have high potential in the prevention and treatment of cancer [98]. Suppression of the NF-κB signaling pathway and its related genes by PL was reported in different cancers [83]. Further, an *in vitro* study demonstrated that PL treatment caused ROS-dependent inactivation of the inhibitor of NF-κB kinase subunit beta (IKKβ), which ultimately caused inhibition of the NF-κB signaling pathway in breast cancer cells [100]. Moreover, PL was found to inactivate NF-κB and dysregulate the expressions of NF-κB mediated proteins, thereby inhibiting metastasis in prostate cancer cells [101]. Furthermore, an *in vivo* study of lung cancer showed that PL modulated the components of the NF-κB signaling pathway, and inhibited tumor growth [102]. These studies suggest that PL and its analogs may have high potential in deactivating the NF-κB pathway, which is constitutively expressed in different cancers.

### PL and JAK/STAT3 signaling

JAK/STAT3 signaling pathway is known to regulate the cellular processes involved in cell survival, cell division, invasion, angiogenesis, migration, metastases, chemoresistance, and radioresistance. JAK/STAT signaling is found to be active in different types of human malignancies and promotes tumorigenesis [18, 84, 103–108]. An *in vitro* study on gastric cancer cells has demonstrated that PL efficiently inhibited cell proliferation, invasion, and migration by blocking the JAK1,2/STAT3 signaling pathway [109]. Furthermore, PL was found to distinctly repress STAT3 activity independent of the phosphorylation of JAK2, which is an upstream regulator of STAT3. Further, the downstream regulators of STAT3, such as c-myc, p21, p27, and survivin, were modulated by PL [110]. Another group synthesized a series of PL derivatives and demonstrated that one of the derivatives, namely CG-06, could suppress the activation of STAT3 by directly binding to it and partly through ROS generation. The derivative was found to be more effective than PL [111]. However, further studies are required to decipher the exact mechanism of STAT3 suppression by PL and its analogs which pave the way in developing therapies against different cancers.

### PL and ERK signaling

ERK is an important cell signaling pathway that is mainly associated with the induction of autophagy [112]. The effect of PL on the ERK pathway was studied on biliary cancer cell lines, which revealed that PL treatment resulted in ROS mediated activation of the ERK pathway, which ultimately induced autophagy in these cells [112]. In addition, this compound increased the levels of intracellular ROS and imparted ROS-dependent cell death via stimulation in c-Jun N-terminal kinase (JNK) and ERK levels. Further, suppression of proteasome activity by PL also imparts to cancer cell death [113]. PL also negatively regulates ERK1/2 signaling pathways, thereby suppressing the level of c-Fos in CRC cells [93]. Additionally, PL was shown to inhibit MEK/ERK signaling in a dose and time-dependent manner, leading to CRC cell death [114]. Moreover, PL was shown to modulate the expression of ERK1/2 and induce cytotoxicity in lung cancer cells [91]. As this pathway is expressed in many other cancers, further investigation on the effect of PL on the proteins involved in this pathway is warranted.

### Other targets

Apart from the aforementioned signaling pathways, PL was found to target a wide range of proteins that play a key role in cancer development. For instance, PL regulated the expressions of critical proteins involved in apoptosis such as Bcl-2, Bcl-2 associated X apoptosis regulator (Bax), Bcl-2 associated agonist of cell death (Bad), B-cell lymphoma-extra-large (Bcl-xL), X-linked inhibitor of apoptosis protein (XIAP), poly (ADP-ribose) polymerase (PARP), and caspases [113, 115–117]. PL was also shown to modulate microtubule-associated protein 1 light chain 3 (LC3), which is considered as one of the important markers of autophagy [90, 118]. A handful of studies also suggested that PL-induced G2/M phase cell cycle arrest by acting on the cell cycle regulatory proteins such as cyclin B1, cyclin D1, CDK1, CDK4, CDK6, and PCNA [100]. PL also increased the expression of growth arrest and DNA-damage-inducible alpha (GADD45α) in gastric cancer cells and downregulated the levels of cdc2 to induce cell cycle arrest [119]. Additionally, PL inhibited the invasiveness and metastatic potential of prostate cancer cells by modulating the expressions of interleukin (IL)-6, IL-8, and MMP-9 [101]. In addition, PL was found to downregulate slug and upregulate E-cadherin and inhibited epithelial-mesenchymal transition (EMT) in breast cancer cells [120]. PL was also found to inhibit transforming growth factor-beta (TGF-β)-induced EMT in breast and lung cancer cells by modulating the expressions of E-cadherin, Snail1, and Twist1 [121]. These studies clearly indicate that PL is a multi-targeted agent and can be used to target multiple deregulations in different cancers.

## Anti-cancer activities of PL

**Figure 4.**
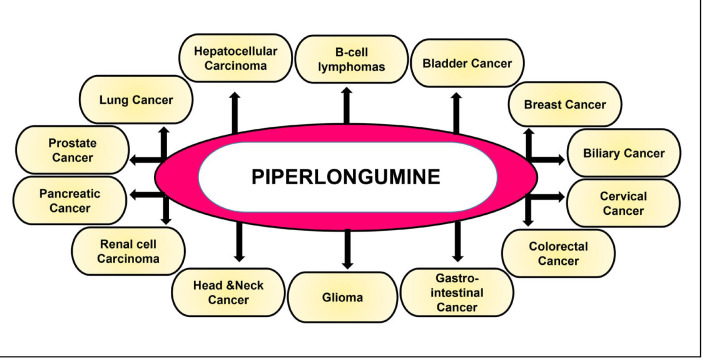
Anti-cancer activity of PL

Increasing lines of evidence suggest that PL has been found to impart potential anti-cancer activities both *in vitro* and *in vivo*. Studies over the years [64, 85, 90–95, 100–102, 109–117, 119–149], have reported the potential of this alkaloid to be used both directly as an anti-cancer drug (Figure 4, Table 1) and in combination with the standard chemotherapeutic drugs (Table 2) to enhance their efficacy. The following section describes the anti-cancer properties of PL in different cancers and its mechanism of action.

**Table 1.** Mechanism of action of PL against different cancers

**Cancer**	***In vitro/In vivo***	**Model**	**Outcome/Mechanism**	**References**
ABC-DLBCL	*In vitro*	OCI-Ly10, U2932, DB ↑p21, ↓NF-κB	↑Apoptosis, ↓Bcl-2, ↓survivin, ↑Bax	[122]
Bladder cancer	*In vitro* *In vivo*	T24, BIU-87, EJ T24 xenograft	G2/M phase arrest, ↓GSH G2/M phase arrest, ↓β-catenin, ↓ ZEB1, ↓N-cadherin, ↓claudin-1, ↓ZO-1, ↓Slug	[123]
Biliary cancer	*In vitro*	HuCCT-1, OCUG-1	G2/M phase arrest, ↑apoptosis, ↑p-ERK, ↑LC3-II, ROS	[112]
Breast cancer	*In vitro*	MCF-7	↓Akt/mTORC1, ↓GSK-3β, ↓TSC2, ↓4E-BP1, ↓p70S6K, ↑LC3-II, ↑autophagy	[90]
	*In vitro*	MDA-MB-231, BT-549, Hs578T	Growth, ↓metastasis, ↓EMT, ↓ZEB1, ↓slug, ↑E-cadherin, ↓MMP2, ↓MMP9, ↑miR-200c, ↓IL-6, ↑ROS, ↑autophagy	[120]
	*In vitro*	MDA-MB-468, MCF-7	↓Proliferation, ↑G2/M arrest, ↑apoptosis, ↓topoisomerase II, ↑p53, ↑p21, ↓Bcl-2, ↑Bax, ↑Cyt C, ↑caspase-3, ↑caspase-7, ↑caspase-8	[116]
	*In vitro*	BT474, MCF7, SkBr3	↓p-HER1, ↓p-HER2, ↓p-HER3, ↑ROS	[126]
	*In vitro*	MCF-7, MCF-10A	↑Apoptosis, ↑HO-1, ↑Nrf2	[213]
	*In vitro*	MCF-7	↑BIM, ↑cleaved caspase-9 and caspase-3, ↓p-FOXO3A, ↓p-Akt	[92]
	*In vivo*	MCF-7 xenograft	↓tumor growth, ↓p-FOXO3A, ↑BIM	[92]
	*In vitro*	SKBR3	↑apoptosis, ↑ROS, ↓Sp1, ↓Sp3, ↓Sp4, ↓cMyc, ↓EGFR, ↓survivin, ↓cMET	[125]
	*In vitro*	MCF-7	↓SETDB1, ↑FosB, ↑cleaved PARP, ↑caspase-9	[117]
	*In vitro*	MCF-7	↑E-cadherin, ↓snail1, ↓Twist1, ↓cyclin D1	[121]
	*In vitro*	MCF-7	↓CDK1, G2/M phase arrest ↓CDK4, ↓CDK6, ↓PCNA, ↓p-CDK1, ↑cyclin B1, ↑ROS, ↓GSH, ↓p-IκBα, ↓mRNA expression of cyclin B1, ↑mRNA p21 expression, ↓NF-κB activation	[100]
Cervical cancer	*In vitro*	HeLa	↑BIM, ↑cleaved caspase-9 and caspase-3, ↓p-FOXO3A, ↓p-Akt	[92]
CCA	*In vitro*	KKU-055, KKU-100, KKU-139, KKU-213, KKU-214	G2/M phase arrest, ↑apoptosis, ↑ROS, ↓p-Akt, ↑Bad, ↓Bcl-2, ↑NQO1, ↑HO-1, ↑SOD2, ↑p21, ↑p-ERK, ↑p-JNK,	[113]
CRC	*In vitro*	LOVO, SW480, HCT116, HT29, HCT8, SW620	↓Cell viability, ↓clonogenic potential, ↓cyclin D1, ↓c-Fos, ↓p-EGFR^Tyr1068^, ↓Akt, ↓ERK1/2	[93]
	*In vivo*	HT-29-xenograft	↓Tumor growth, ↓c-Fos and cyclin D1 positive cells	[93]
	*In vitro*	SW-620	↓Proliferation	[116]
	*In vitro*	HCT 116, HCT 116 Bax^−/−^, HCT 116 p21^−/−^, HCT 116 p53^−/−^	G2/M phase arrest, ↑apoptosis	[131]
	*In vivo*	DMH & DSS induced	↓Tumor growth, G2/M phase arrest, ↑apoptosis, ↓Bcl-2, ↑cleaved caspase-3, ↓Ras, ↓cyclin D1, ↓NF-κB	[130]
	*In vitro*	DLD-1	G2/M cell cycle arrest, ↑apoptosis, ↑ROS, ↓GSH, ↓TrxR	[129]
	*In vitro*	HT29, SW620, HCT116	↑Nrf2, ↑ROS, ↑Bax, ↑cleaved caspase-3, ↑cleaved PARP, ↑MDM2	[132]
	*In vivo*	HT-29-xenograft	↓Tumor growth, ↓tumor volume, ↓mutant p53, ↑Bax, ↑cleaved PARP	[132]
	*In vitro*	HT-29, HCT 116	↑Apoptosis, ↑p-ERK	[114]
	*In vivo*	AOM/DSS induced	↓COX-2, ↓IL-6, ↓β-catenin, ↓snail	[128]
	*In vitro*	HCT116	↑apoptosis, ↑p-c-Jun, JNK activation	[133]
	*In vitro*	INT-407, HCT-116	↑ROS, ↓FN1, ↓CDH2, ↓CTNNB1, ↓Bcl2, ↓survivin, ↑p53, ↑Bax, ↑SMAD4, ↑p21, ↓Twist	[127]
Gastric cancer	*In vitro*	AGS, HGC-27	G2/M phase arrest, ↑GADD45α, ↑ROS	[119]
	*In vitro*	SGC-7901, BGC-823	↑ROS, ↓MDM-2, ↓cyclin B1, ↓Cdc2, G2/M phase arrest, ↑p-eIF2α, ↑ATF4, KATO III ↑CHOP, ↑apoptosis	[135]
	*In vivo*	SGC-7901 xenograft	↑ROS, ↓TrxR1, ↑cleaved caspase-3, ↑CHOP, ↑MDA	[135]
	*In vitro*	MKN45, AGS	G2/M phase arrest, ↓p-JAK1, ↓p-JAK2, ↓p-STAT3, ↓Ki-67, ↓MMP-9, ↓Twist, ↓cyclin D1	[109]
	*In vitro*	MGC-803	↑BIM, ↑cleaved caspase-9 and caspase-3, ↓p-FOXO3A, ↓p-Akt	[92]
Glioma	*In vitro*	HGG	↑ROS, ↓PRDX4, ↑cleaved caspase-3, ↑P-H2AX, ↑CHOP, ↑p-eIF2α, ↑apoptosis	[136]
	*In vitro*	U87MG	↓Proliferation, ↑apoptosis, ↑FOS, ↑RAF1, ↑NFKB1, ↑NFKB1A, ↑NFKB2, ↑PIK3CA, ↑TP53, ↓AKT1, ↓AKT2, ↓DVL1, ↓EGFR, ↓PIK3R1, ↑PTEN, ↑BRAF, ↓KRAS	[134]
HNC	*In vitro*	UMSCC1, UMSCC10A, UMSCC17A	↑Apoptosis, ↑LC3-II, ↑ROS, ↑8-oxo-dG, ↓GSTP1 activity	[142]
	*In vivo*	UMSCC10A xenograft	↓Tumor volume	[142]
	*In vitro*	SAS, CGHNC8	↓SOX2, ↓NANOG, ↓Oct-4, ↑E-cadherin, ↑CK18, ↓N-cadherin, ↓vimentin, ↓snail, ↓slug	[144]
	*In vivo*	SAS & CGHNC8 xenograft	↓Tumor weight, ↓tumor growth	[144]
	*In vitro*	OC2, OCSL	↓Proliferation, ↑G0/G1 arrest, ↑p21, ↑apoptosis, ↑PARP-1, ↑caspase-3, ↑senescence	[145]
HCC	*In vitro*	HUH-7, HepG2	↑ROS, ↓proliferation, ↑apoptosis, ↓procaspase-3, ↑Bax, ↑cleaved caspase-3, ↑G2/M arrest, ↑ATF4, ↑p-eIF2α, ↑p-PERK, ↓TrxR1, ↓Bcl-2	[64]
	*In vivo*	HUH-7 xenograft	↓Tumor volume and weight, ↑ROS, ↓TrxR1	[64]
Lung cancer	*In vitro*	A549	↓Proliferation, ↑cell death, ↓migration	[143]
	*In vitro*	A549, H1299	↑Apoptosis, ↑ROS	[138]
	*In vitro*	H1975, H23, HCC827	↓HK2, ↑cleaved-PARP, ↑caspase-3	[94]
	*In vivo*	H1975 & HCC827 xenograft	↓p-Akt, ↓p-S6, HK2, ↓Ki-67, ↓tumor weight, ↓tumor growth	[94]
	*In vitro*	A549, A549/DTX	↑Cleaved PARP, ↓Bcl-2, ↑Bax, ↑LC3-II, ↑apoptosis, ↓p-Akt (Thr308 and Ser473), ↓PI3K, ↓mTOR (Ser2448)	[95]
	*In vivo*	A549/DTX xenograft	↓Tumor volume, ↓Ki-67, ↓p-Akt, ↓mTOR	[95]
	*In vitro*	A549	↑ROS, ↑LC3B-II	[137]
	*In vitro*	A549	↑Apoptosis, ↑ROS, ↓Sp1, ↓Sp3, ↓Sp4, ↓cMyc, ↓EGFR, ↓survivin, ↓cMET	[125]
	*In vitro*	A549, NCI-H460	↑Apoptosis, ↑Bax, ↑cleaved caspase-3 and -8, ↓Bcl-2	[102]
	*In vivo*	A549 xenograft	↓Tumor volume, ↓tumor weight, ↑Fas, ↑DR4, ↑Bax, ↓Bcl-2, ↑cleaved caspase-3 and -8, ↓nuclear p50 and p65	[102]
	*In vitro*	A549	↑E-cadherin, ↓snail1, ↓Twist1	[121]
	*In vitro*	A549	G1 phase arrest, ↑ROS, ↓cyclin D1, ↓CDK6, ↑p-ERK1/2, ↓p-Akt, ↓p-Rb, ↓NF-κB p65 nuclear translocation, ↓Rb, ↓CDK4	[91]
MM	*In vitro*	OPM2, MM1R, U266, IM-9, NCI-H929	↑Apoptosis, ↑caspase-3, -9, or -8 activity, ↑cyclin E, ↓Bcl-2, ↑Bax/Bcl-2, ↑ROS, ↓STAT3 activity, ↑p21, ↑p27, ↓c-myc, ↓cyclin A, ↓survivin	[110]
Prostate cancer	*In vitro*	PC-3, DU-145, LNCaP	↓Proliferation, ↓NF-κB, ↓IL-6, ↓IL-8, ↓MMP-9, ↓invasion, ↓adhesion, ↓ICAM-1	[101]
	*In vitro*	DU-145	↓p-STAT3^Tyr-705^, ↓cyclin A, ↓survivin, ↑cleaved PARP, ↓cell survival, ↑G1/S arrest, ↑ERK1/2, ↓Bcl-2	[111]
	*In vitro*	PC-3	↓Akt/mTORC1, ↓GSK-3β, ↓TSC2, ↓p70S6K, ↑LC3-II, ↑autophagy, ↓4E-BP1	[90]
Pancreatic cancer	*In vitro*	MIAPaCa-2, PANC-1	↑Cell death, ↑ROS	[140]
	*In vitro*	Panc1, L3.6pL	↑Apoptosis, ↑ROS, ↓Sp1, ↓Sp3, ↓Sp4 ↓cMyc, ↓EGFR, ↓survivin, ↓cMET	[125]
	*In vitro*	PANC-1, MIA PaCa-2	↑ROS, ↑SOD1, ↑GSTP1, ↑HO-1	[85]
	*In vivo*	PANC-1 xenograft	↓Tumor growth, ↓Ki67, ↑8-OHdG	[85]
	*In vitro*	PANC-1, AsPC-1, BxPC-3	↓c-Myc, ↓cyclin D1, ↓Bcl-2, ↓Bcl-xL, ↓XIAP, ↓VEGF, ↓MMP-9, ↓NF-κB, ↓survivin,	[115]
	*In vivo*	BxPC-3 xenograft	↓Tumor growth, ↓c-Myc, ↓cyclin D1, ↓Bcl-2, ↓survivin, ↓XIAP, ↓VEGF, ↓MMP-9, ↓NF-κB, ↓Bcl-xL	[115]
	*In vitro*	MIA PaCa-2, PANC-1	↓GST activity, ↑JNK activation, ↑c-Jun, ↑HMOX1, ↑HSPA1A, ↑Myc, ↑CASP3, ↑PIK3CG, ↓Bcl-2, ↓NF-κB1, ↓AKT3, ↑cleaved caspase-3, ↑cleaved PARP, ↑apoptosis, ↑ATF-2, ↑CDKN1A, ↓p-ERK	[139]
RCC	*In vitro*	786-O	↓Akt/mTORC1, ↓GSK-3β, ↓TSC2, ↓p70S6K, ↑LC3-II, ↓4E-BP1	[90]
	*In vitro*	786-O	↑Apoptosis, ↑ ROS, ↓Sp1, ↓Sp3, ↓Sp4, ↓cMyc, ↓EGFR, ↓survivin, ↓cMET	[125]
	*In vitro*	786-O, PNX0010	↓cMET, ↓p-ERK1/2, ↓p-STAT3, ↓p-Akt, ↑ROS	[146]
	*In vivo*	PTX xenograft	↓Tumor growth, ↓cMET	[146]
Skin cancer	*In vitro*	A375, A875, B16-F10	G2/M phase arrest, ↑apoptosis, ↑cleaved caspase-3, ↓Bcl-2, ↑Bax, ↑p-JNK, ↑ROS, ↑p21, ↑p27	[141]

8-OHdG: 8-hydroxy-2’-deoxyguanosine; ABC-DLBCL: activated B cell-like subtype of diffuse large B cell lymphoma; ATF4: activating transcription factor-4; CCA: cholangiocarcinoma; Cdc: cell division control; CHOP: C/EBP homologous protein; CK18: cytokeratin 18; cMET: hepatocyte growth factor receptor; COX-2: cyclooxygenase-2; DMH: 1,2-dimethylhydrazine; DR4: death receptor 4; DSS: dextran sulfate sodium; DTX: docetaxel-resistant; EGFR: epidermal growth factor receptor; FOXO3A: forkhead box O3A; GST: GSH S-transferase; GSTP1: GST pi 1; HCC: hepatocellular carcinoma; HO-1: heme oxygenase-1; MDA: malondialdehyde; MM: multiple myeloma, MMP-9: matrix metalloproteinases-9; Nrf2: nuclear factor-erythroid-2-related factor-2; HK2: hexokinase 2; NQO1: NAD(P)H quinone dehydrogenase 1; p-eIF2α: phosphorylation of eukaryotic initiation factor-2α; PRDX4: peroxiredoxin 4; PTX: paclitaxel; Rb: retinoblastoma; RCC: renal cell carcinoma; SETDB1: SET domain bifurcated histone lysine methyltransferase 1; SOD: superoxide dismutase; SOX2: sex determining region Y-box 2; Sp: specificity protein; TrxR: thioredoxin reductase; VEGF: vascular endothelial growth factor; ZEB1: zinc finger E-box binding homeobox 1; ZO-1: zonula occludens-1

**Table 2.** Chemosensitizing potential of PL

**Drugs**	**Cancer**	***In vitro*/*In vivo***	**Model**	**Mechanism**	**References**
Bortezomib	MM	*In vitro*	NCI-H929	↑Apoptosis, ↓p-STAT3	[110]
Cisplatin	HNC	*In vitro*	AMC-HN2, -HN3, -HN4, -HN6, -HN7, -HN8, SNU-1041, -1066, -1076, HN30, HN31, UMSCC1, 93-VU-147T	↑ROS, ↓GSH, ↑GSSG, ↑PUMA, ↑cleaved PARP, ↑p-JNK, ↓GSTP1, -HN9, ↑p-p53 (Ser 15), ↑apoptosis	[149]
		*In vivo*	AMC-HN2 & -HN9, xenograft	↓Tumor growth, ↑p53, ↑apoptosis	[149]
Doxorubicin	Prostate cancer	*In vitro*	DU-145	↑Apoptosis, ↑caspase-3, ↑cleaved PARP	[148]
5-Flurouracil	Oral cancer	*In vitro*	SAS, CGHNC8	↓Cell viability, ↓survival	[144]
Gemcitabine	Pancreatic cancer	*In vitro*	BxPC-3, PANC-1, AsPC-1	↑Apoptosis, ↓NF-κB	[115]
		*In vivo*	BxPC-3 xenograft	↓Tumor burden, ↑apoptosis, ↓NF-κB	[115]
Oxaliplatin	Gastric cancer	*In vitro*	SGC-7901, AGS, BGC-823	↓TrxR1 activity, ↑ROS, ↑apoptosis, Activation of p38 and JNK signaling pathways, ↑γ-H2A.X	[147]
		*In vivo*	SGC-7901 xenograft	↓Tumor growth, ↓TrxR1 activity	[147]
PTX	Intestinal Cancer	*In vitro*	INT-407 and HCT-116	↓Proliferation	[127]

GSSG: GSH disulphide; p: phosphorylated

### ABC-DLBCL

Diffuse large B cell lymphoma (DLBCL) represents the most typical type of non-Hodgkin’s lymphoma, and ABC-DLBCL is the most aggressive form of DLBCL, which results in poor 5-year survival of patients [122, 150]. It was reported that PL significantly induced apoptosis and cell death in ABC-DLBCL cell lines via suppression of NF-κB signaling pathway and modulating the NF-κB-mediated proteins responsible for apoptosis and cell survival such as Bcl-2, survivin, Bax, and p21 [122].

### Bladder cancer

Bladder cancer, one of the most predominant cancers of the urinary tract, occurring most frequently in males than in females, is found to affect around 430,000 people worldwide annually [151]. PL was found to inhibit the proliferation, migration, and invasion of bladder cancer cells *in vitro* by targeting the F-actin reorganization and modulating the ERK and PKC pathways. It further arrested the cell cycle at the G2/M phase. The results were also confirmed through *in vivo* studies where PL was demonstrated to inhibit tumor growth and EMT, one of the important hallmarks of cancer [123].

### Biliary cancer

Biliary cancer is one of the most aggressive types of neoplasms, with a very high rate of mortality, poor prognosis, and low 5-year survival rate [152]. PL was reported to show potent anti-proliferative activity against both biliary epithelial tumor cells and gallbladder carcinoma cells *in vitro* via arresting the cell cycle at the G2/M and G0/G1 phase, respectively. Further, PL induced apoptosis and autophagy in biliary cancer cell lines, which could be attributed to the modulation of the ROS-activated ERK pathway [112].

Another rare type of biliary cancer, CCA, often results in poor diagnosis and prognosis [59, 153]. Studies assessing the efficacy of PL treatment on CCA cell lines have revealed that this compound induced cell cycle arrest at G2/M phase and apoptosis by regulating the expression of different pro- and anti-apoptotic proteins [113]. In addition, this compound increased the levels of intracellular ROS and imparted ROS-depended cell death via stimulation of JNK and ERK levels. Further, the suppression of proteasome activity by PL was also suggested to be responsible for inducing apoptosis [113].

### Breast cancer

Breast cancer is one of the most prevalent cancers worldwide and constitutes the major cause of cancer-related mortality in females [154–156]. Studies over the years [90, 92, 100, 116, 117, 120, 121, 125, 126] have demonstrated that PL regulated the major processes leading to the development and progression of breast cancer, including cell proliferation, growth, invasion, migration, metastasis, and EMT. An *in vitro* study has demonstrated that PL treatment induced apoptosis and autophagy in breast cancer cells via modulating the downstream components of the Akt/mTOR signaling pathway [90]. Another study reported that PL suppresses TGF-β induced migration, invasion, and EMT in breast cancer cells by reversing the effects of TGF-β on the EMT-related protein E-cadherin and modulating the expressions of Snail1 and Twist1 [121]. Additionally, PL was found to inhibit proliferation and induce apoptosis in breast cancer cells by modulating the expressions of critical proteins such as topoisomerase II, p53, p21, Bcl-2, Bax, cytochrome c (Cyt C), caspase-3, caspase-7, and caspase-8 [116]. Additionally, studies have also demonstrated that PL suppressed the invasiveness of triple-negative breast cancer cells (TNBC) and inhibited EMT through modulating the expressions of key proteins such as MMPs, ZEB1, Slug, and E-cadherin. Further, it enhanced the expression of the microRNA, miR-200c, loss of which plays a critical role in tumorigenesis [120, 124]. Furthermore, PL was found to target the human epidermal growth factor receptor (HER) family in breast cancer, which plays a major role in controlling the intracellular signaling pathways. PL significantly diminished the phosphorylated levels of HER1, HER2, and HER3 via increasing the generation of ROS in breast cancer cells [126]. It was also demonstrated that ROS dependent cytotoxicity exerted by PL could suppress the expression of IKKβ, which resulted in inactivation of the NF-κB signaling pathway and subsequently an upsurge in the levels of p21 mRNA [100]. Additionally, another study reported that PL suppressed SETDB1, which induced the level of caspase-9 dependent-PARP cleavage leading to apoptosis in MCF7 cells. In addition, PL also enhanced the transcriptional activity of FBJ murine osteosarcoma viral oncogene homolog B (*FosB*), which might also be responsible for PL-induced apoptosis [117].

### Cervical cancer

Cervical cancer is the fourth most prevalent cancer among women, and it constitutes about 4% of all the malignancies [157, 158]. PL was shown to induce apoptosis and inhibit cell viability in cervical cancer cell line HeLa. In addition, it was observed that PL upregulated the expression of pro-apoptotic protein Bcl-2-like protein 11 (BIM) and significantly inhibited the Akt signaling pathway, thereby leading to the dephosphorylation of FOXO3A [92]. However, further in-depth studies are required to establish the role of PL in this cancer.

### CRC

The incidence of CRC is increasing globally, and it is also estimated that by 2035, the percentage of mortality due to CRC will increase by 60-70% [50, 159, 160]. Numerous studies have demonstrated the efficiency of PL against CRC both *in vitro* and *in vivo*. One such study has reported that PL induced cytotoxicity on CRC cells mainly through the suppression of cyclin D1, which was involved in maintaining the tumorigenicity of the CRC cells. PL also negatively regulated the Akt and ERK1/2 signaling pathways, thereby suppressing the levels of *c-Fos* in CRC cells. Another *in vivo* study by the same group has demonstrated that PL significantly inhibited the tumor growth in the xenograft mouse model of CRC [93]. Further, a study has also proposed that PL inhibited mitogen-activated protein kinase (MAPK)/ERK kinase (MEK) signaling in CRC cells, thereby inducing cell death in a dose and time-dependent manner [114]. Further, PL was shown to significantly inhibit the proliferation of SW-620 CRC cell lines [116]. Moreover, PL was found to induce apoptosis in CRC cells by inhibiting the JNK signaling pathway [133]. In addition, this compound was able to induce cytotoxicity and apoptosis in CRC cells without alerting the expressions of Bax, p21, and p53 [131]. PL was also reported to induce ROS generation in CRC cells and target the GSH anti-oxidant and TrxR systems. Further, enhanced levels of ROS generated by PL led to DNA damage and cell cycle arrest in the CRC cells [129].

The potential of PL was also examined in a DMH/DSS-induced experimental colon cancer model. On PL treatment, a significant improvement in weight and food intake of the mice was observed. In addition, cell cycle arrest at the G2/M phase and induction of apoptosis were observed, which resulted in the reduction of tumor growth. Further, the potential of PL in inhibiting tumor formation was attributed to the inhibition of the Ras/PI3K/Akt/mTOR signaling pathway [130]. This compound was also found to suppress tumor growth in a nude mouse model and induced the restoration of wild-type p53 function [132]. Additionally, administration of PL could significantly alleviate the levels of COX-2, IL-6, β-catenin, and snail, thereby attenuating inflammation and tumor progression in azoxymethane (AOM)/ DSS-induced mouse model of CRC [128]. PL was also found to impart morphological changes and nuclear damage in CRC, which further prompted apoptosis and cell death. Apart from this, PL drastically increased the intracellular levels of ROS, which also resulted in apoptosis, as evident from the modulation in the expression of numerous proteins such as Bax, Bcl-2, survivin, p53, and p21 associated with the process. Additionally, PL was found to hamper the migration potential of the CRC cells [127].

### Gastric cancer

Gastric cancer may occur from acute gastritis and is known to be the third prevalent cause of cancer-related mortalities worldwide [161–163]. Over the years, the potential of PL against gastric cancer is demonstrated with the help of numerous pre-clinical studies. For instance, PL was reported to induce the generation of ROS in gastric cancer cells, which resulted in inhibition of cell proliferation and subsequently cell death. Further, induction of GADD45α was observed upon PL treatment which led to cell cycle arrest in the G2/M phase [119]. It was also suggested that PL might modulate the expression of TrxR1, one of the key anti-oxidant enzymes, both *in vitro* and *in vivo*, which caused ROS-mediated apoptosis in gastric cancer cells [135]. Moreover, another study has proposed the anti-cancer potential of PL might be attributed to its ability to inhibit the JAK1,2/STAT3 signaling pathway [109]. Further, PL was found to induce BIM-mediated apoptosis regulated by the significant upregulation of the tumor suppressor and transcription factor FOXO3A [92].

### Glioma

High-grade glioma (HGG) is the commonest type of brain cancer out of the 120 types, which results in a poor prognosis [136, 164]. PL was shown to interact with the levels of ROS-degrading enzyme PRDX4 and induce ROS generation in HGG cells, which further led to the induction of endoplasmic reticulum (ER) stress and apoptosis [136]. Glioblastoma multiforme (GBM) is the most prevalent and lethal type of glioma with a poor 5-year survival rate [59, 136]. A potent analog of PL, (*E*)-*N*-(4-fluorobenzyl)-3-(3,4,5-trimethoxyphenyl) acrylamide (NFBTA), showed significant anti-cancer activity against GBM cells. *In vitro* studies on NFBTA treated GBM cell line U87MG have also reported that this compound is highly selective towards the cancer cells and imparted significant anti-proliferative and apoptotic effects. Further, NFBTA was also involved in modulating the expression of the key factors of the oncogenic signaling pathways such as *FOS*, *RAF1*, *NFKB1/1A/2*, *BRAF*, *PIK3CA/R1*, Tumor protein 53 (*TP53*), phosphatase and tensin homolog (*PTEN*), *Akt1/2*, *EGFR*, dishevelled segment polarity protein 1 (*DVL1*), and *KRAS* [134].

### Head and neck cancer

Head and neck cancer (HNC) is a frequently occurring malignancy globally is associated with poor prognosis [165]. In a pre-clinical study, the effects of a combination of PL and p53-reactivation and induction of massive apoptosis-1 (PRIMA-1^Met^, also known as APR-246) on HNC cells were evaluated. PRIMA-1^Met^ is known to reinstate the DNA-binding ability of mutant p53 and restore the wild-type p53 activity. The combination was found to significantly induce apoptosis and autophagy in HNC cells and reduce tumor growth in animal models and thus could be considered as a novel treatment strategy for HNC [142]. Oral cancer, the commonest form of HNC, causes around 128,000 deaths yearly and is one of the most prevalent in Southern Asia and the Pacific islands [89, 166]. Several pre-clinical studies have evidenced the potential of PL against oral cancer. For instance, an *in vitro* study has reported that PL helped in suppressing the stemness of oral cancer cells, an important property needed for tumor maintenance, by modulating the levels of the transcription factors Oct-4, NANOG, SOX2, and CK18. This compound also significantly suppressed the critical hallmarks of cancer such as migration, invasion, and EMT, thereby inhibiting tumor growth both *in vitro* and *in vivo* [144]. In addition, another *in vitro* study has revealed that PL could also bring about senescence via upregulation of p21, which is known to be involved in numerous biological processes, including senescence [145].

### HCC

HCC stands as the fifth most prevalent type of cancer among males and seventh among females [167, 168]. *In vitro* studies on liver cancer cell lines have reported that upon treatment with PL, the ROS levels increased significantly, which exerts anti-proliferative effects on these cells. Further, PL treatment led to ROS-mediated apoptosis, G2/M phase cell cycle arrest, and ER stress in the liver cancer cells. Additionally, the effects of PL treatment were also evaluated in *in vivo* models, where a decrease in the tumor progression was observed. The tumor-suppressive role of PL was mainly attributed to its ability to target TrxR1, a key enzyme of the anti-oxidant system, which was found to be highly upregulated in the case of liver cancer [64].

### Lung cancer

Lung cancer is one of the most commonly occurring cancers and has the highest rate of mortality worldwide [168–170]. An *in vitro* study analyzed the effects of PL in lung cancer cell lines with a modulated expression of profilin-1 (PFN1), one of the actin-binding proteins (ABPs), which plays a critical role in the regulation of cellular migration. It was observed that PL could significantly exert cytotoxic effects on the cancerous cells and its effects were more prominent when the expression of PFN1 was downregulated [143]. Another preclinical study on non-small cell lung carcinoma (NSCLC) cells revealed that PL treatment decreased the levels of HK2, an enzyme of the glycolysis process which was found to be involved in tumor progression. Subsequently, the glycolysis process of the cancerous cells was dysregulated by PL treatment. Further, it was put forth that inhibition of Akt phosphorylation by PL was partially responsible for the inhibition of glycolysis and induction of apoptosis in lung cancer cells. The results were further validated in animal models of lung cancer which presented similar outcomes [94]. Additionally, the efficacy of PL in inducing apoptosis and autophagy in both *in vitro* and *in vivo* models of lung cancer was attributed to its role in suppressing the components of the PI3K/Akt/mTOR signaling pathway [95]. Moreover, PL was shown to suppress the activation of Akt in lung cancer cells which further inhibited the expressions of ERK 1/2 and NF-κB [91].

Another *in vitro* study has reported that in addition to inducing ROS mediated cytotoxicity in lung cancer cells, PL modulated the expressions of the Sp regulated genes such as *cyclin D1*, EGFR, hepatocyte growth factor receptor (*HGFR*), and *survivin* and also suppressed the transcription factors Sp1, Sp3, and Sp4 [125]. Furthermore, PL was found to act upon the components of the NF-κB signaling pathway and inhibited tumor progression both *in vitro* and *in vivo* [102]. Also, as observed in breast cancer cells, PL was found to be effective in inhibiting TGF-β-induced EMT and invasion in lung cancer cells. This compound exerted similar activity by reversing the effects of TGF-β on the EMT-related protein E-cadherin and interrelating the expressions of Snail1 and Twist1 [121].

PL was also found to impart cytotoxic effects on NSCLC cells compared to the normal lung cells both alone and synergistically when used in combination with another alkaloid, sanguinarine [138]. This compound also induced anti-cancer activity by promoting the levels of ROS and 1B-LC3B-II, an essential protein involved in autophagy. However, PL in combination with gemcitabine enhanced the cytotoxicity but failed to upregulate the levels of ROS and LC3B-II [137].

### MM

MM is a malignancy of the B-cells, characterized by an atypical growth and invasion of plasma cells to the bone marrow [171, 172]. The potential of PL against MM was investigated by an *in vitro* study where PL was found to exert anti-proliferative and anti-apoptotic effects in MM cells. The anti-apoptotic effects of PL were mediated by its ability to modulate the Fas- and mitochondria-dependent pathways. Following PL treatment, the expression of Bcl-2 was diminished, and an inclination in the Bax/Bcl-2 ratio was observed. Further, activation of the caspase family of proteins was also increased. Moreover, significant inhibition of the STAT3 signaling pathway was also evidenced after PL treatment [110].

### Prostate cancer

Prostate cancer is a very common cancer in men with a high rate of incidence in the Western countries as compared to the Asian population [97, 173, 174]. PL was found to significantly inhibit the activation of NF-κB and modulate the expressions of NF-κB mediated proteins such as IL-6, IL-8, MMP-9, and intercellular adhesion molecule 1 (ICAM-1), thereby suppressing the metastatic potential of the prostate cancer cells [101]. In addition, PL was shown to inactivate the Akt/mTOR signaling via ROS generation, which subsequently induced cell death and autophagy in prostate cancer cells. The same study also evidenced the inhibition of tumor growth in a mouse model of prostate cancer by both PL alone and in combination with chloroquine [90]. Moreover, an *in vitro* study on PL derivatives demonstrated that one of the derivatives, namely CG-06, could suppress the activation of STAT3 by directly binding to it and partly through ROS generation more effectively than PL [111].

### Pancreatic cancer

Pancreatic cancer is a lethal disease with a poor prognosis [26, 175, 176]. A recent study evaluating the effect of PL on pancreatic cancer cells has reported that this compound could induce ferroptosis via ROS generation. The study further displayed that the cytotoxic potential of PL against pancreatic cancer cells was greatly enhanced when used in combination with cotylenin A, a growth regulator, and the commercial drug sulfasalazine [140]. Additionally, Karki et al. [125], who have evaluated the potential of PL in breast and lung cancers, have reported that the compound was effective in pancreatic cancer cells as well, mainly through the suppression of the Sp transcription factors and their regulated genes. Also, PL was found to inhibit cell growth via inducing ROS-mediated DNA damage both *in vitro* and *in vivo* models of pancreatic cancer [85]. Furthermore, PL suppressed NF-κB activation, and other NF-κB regulated genes including c-myc, cyclin D1, Bcl-2, Bcl-xL, survivin, XIAP, VEGF, and MMP-9 in pre-clinical models of pancreatic cancer, which led to suppression of cell proliferation and induction apoptosis [115]. Additionally, PL treatment resulted in the activation of the JNK signaling pathway and time-dependent activation of ERK signaling in pancreatic cancer cells, thereby imparting apoptotic cell death [139].

### RCC

RCC is the most common type of kidney tumor, which has a high rate of incidence in men than in women [177, 178]. Studies on RCC cells have proved that PL could significantly suppress the Akt/mTOR signaling pathway mainly through generating ROS in RCC cells, which subsequently led to cell death and inhibition of critical hallmarks associated with tumor initiation and progression [90]. Furthermore, Karki et al. [125] have evaluated the potential of PL in inhibiting RCC and reported that this compound suppresses the Sp transcription factors and regulated genes. Additionally, PL was shown to inhibit the expression of c-Met through ROS-mediated proteasome independent pathway in RCC cells, which subsequently inhibited the phosphorylated levels of ERK1/2, STAT3, and Akt. The analogs of PL, namely, PL-fluorophenyl (PL-FPh) and PL-Dimer (PL-Di), were further found to impart more prominent anti-tumor effects both *in vitro* and *in vivo* as compared to the native form of PL [146].

### Skin cancer

Skin cancer is the most prevalent cancer type among Caucasians, which can be of two types, namely, melanoma and non-melanoma [179, 180]. The potential of PL was evaluated in melanoma cells, where this compound was found to induce cytotoxicity in a concentration and time-dependent manner. This compound induced ROS generation, which ultimately led to a decline in the mitochondrial membrane potential. Further, PL was shown to modulate the expression of p21, p27, caspases-3, Bax/Bcl-2, and JNK, which are the critical regulators involved in proliferation, cell apoptotic death, and JNK signaling pathway [141].

## Chemosensitizing potential of PL

Chemoresistance stands as the major constraint over using the standard chemotherapeutic agents available for cancer [11, 12, 14]. Over the years, several studies have evaluated the potential of PL as a potent and affordable anti-cancer drug. PL was found to modulate the key components of the critical signaling pathways which are involved in developing chemoresistance in cancer cells. Thus, the recent focus was driven towards developing PL as a chemosensitizer which sensitized the cancer cells towards the commercially available chemotherapeutics. The following section describes the role of PL in chemosensitizing the cancer cells towards some of the essential drugs.

### Cisplatin

Cisplatin is a very common drug that is used in the treatment of many cancer types, including ovarian cancer and HNC [181]. However, cisplatin treatment was known to induce chemoresistance in cancer cells. Over the years, numerous studies have suggested that cisplatin resistance could arise due to epigenetic changes, including lesser accumulation of the platinum compounds in the cells, detoxification by GSH conjugates, metallothioneins, and various other antioxidants, rise in the levels of DNA damage repair, changes in the status of DNA-methylation, upregulated expression of chaperones, modulation of microRNA expression, transcription factors and small GTPases, and dysregulation of the apoptosis and EMT pathway [182]. In addition, it was observed that loss or mutation of p53 in HNC is linked to cisplatin resistance due to suppression of senescence [183]. However, a recent study showed that PL was able to reduce the cisplatin resistance in p53 mutant HNC cells both *in vitro* and *in vivo*. *In vitro*, the combination of PL and cisplatin imparted cytotoxicity synergistically and induced ROS generation, and the expression of p53 and p-p53, and cleaved PARP, thereby leading to apoptosis. Similar results were obtained in *in vivo* studies as well, where significant apoptotic death and inhibition of tumor growth were observed [149].

### Doxorubicin

Doxorubicin, another widely used anti-cancer drug that has been employed for the treatment of a wide variety of cancers such as breast, gastric, lung, lymphoma (Hodgkin’s and non-Hodgkin’s), MM, ovarian, sarcoma, and thyroid. However, treatment of cancer cells with doxorubicin is known to induce chemoresistance through the modulation of different signaling pathways, non-metabolic pathways, and post-translational modifications [184, 185]. Numerous reports have suggested that carbonyl reductase 1 (CBR1), which results in a declined biotransformation of anthracyclines to lesser active metabolites, might be a key target for the chemosensitizing agents. It was reported that when doxorubicin was used in combination with PL, the formation of the inactive metabolite doxorubicinol (DOXol) was reduced. Further, molecular modeling studies have suggested the interaction between PL and the active sites of CBR1 is similar as reported in the case of the previously studied CBR1 inhibitors, which showed potential chemosensitizing effects. Additionally, PL was evidenced to suppress chemoresistance of prostate cancer cells and sensitize the cancer cells to doxorubicin. When doxorubicin was administered in combination with PL in prostate cancer cells, the effect was synergistic, and the treatment induced apoptosis, as evident from the modulated expression of the apoptotic proteins such as caspase-3 and PARP. These effects might be attributed to the ability of PL to bind with and inhibit CBR1 [148, 186].

### 5-Fluorouracil

5-Fluorouracil (5-FU) has been in use since 1957 and is still the third most frequently used anti-cancer drug for the treatment of solid tumors in the world [187, 188]. It is a very common drug that is used in the treatment of oral cancer. However, over the years, studies have evinced that extensive use of the drug has led to the development of chemoresistance of oral cancer cells. Studies have shown that high levels of CSC markers in cancer cells also result in the development of chemoresistance in cancer cells [189]. PL was found to significantly inhibit the stem cell properties in oral cancer cells, which might have contributed to the chemosensitizing potential of PL. Thus, PL, when used in combination with 5-FU, helped in enhancing the cytotoxic effects by reducing the cell viability and survival of oral cancer cells [144].

### Gemcitabine

Gemcitabine is the first-line therapy for pancreatic cancer. However, in the majority of the patients, it induces chemoresistance and becomes ineffective due to the activation of the NF-κB pathway, which is predominantly implicated in the development of chemoresistance in this cancer [26]. An *in vitro* study on pancreatic cancer cells has evidenced that gemcitabine, when used in combination with PL, imparted more prominent anti-cancer effects mediated through blockage of NF-κB activation. Furthermore, in *in vivo* studies, the combined treatment was found to be more effective in reducing tumor growth compared to gemcitabine alone [115].

### Oxaliplatin

Oxaliplatin is a platinum-based chemotherapeutic drug that is used for the treatment of various cancers, including colorectal and gastric cancers [147, 190]. However, chemoresistance and severe side-effects associated with the use of this drug limit its efficacy [191, 192]. TrxR1, a flavoenzyme, is found to be overexpressed invarious types ofcancers and is associated with improved tumor growth and chemoresistance. It was observed that PL helped in sensitizing gastric cancer cells towards oxaliplatin mainly by enhancing the generation of ROS via suppressing the activation of TrxR1, thereby inducing apoptosis. This combination also resulted in the activation of the p38 and JNK cell signaling pathways *in vitro* and *in vivo* [147].

### PTX

PTX or taxol is one of the widely used anti-cancer drugs used commonly for the treatment of breast, lung, and ovarian cancers, etc. [193]. Though the drug is found to be very effective, extensive use of it was often found to result in chemoresistance of cancer cells. However, the exact mechanism of PTX associated chemoresistance is still not precise [194]. A recent study showed that PL sensitized intestinal cancer cells to PTX by effectively inhibiting the proliferation of the cancer cells. Additionally, it was elucidated that PL might have activated the SMAD4 pathway, thereby improving the chemotherapeutic effect of the cancer cells by stimulating p21 and its downstream pathways resulting in apoptosis [127].

## Radiosensitizing potential of PL

Radiotherapy is a widely used treatment modality for cancer patients and is considered to have an added advantage due to its localized application. However, over the years, the development of radioresistance due to alterations in the signaling pathways associated with radiosensitivity, tumor heterogeneity and cancer stem cells have limited the use of radiotherapy and resulted in poor prognosis in the patients [195]. Therefore, the application of radiosensitizers was found to be an effective method for improving the radiosensitivity of cancer cells and minimizing the adverse effects of radiotherapy on the adjacent normal cells [196].

Small molecule radiosensitizers, including oxygen, active phytochemicals, hypoxia-specific cytotoxins, and agents, modulate the cell signal pathways involved in radioresistance [197]. Oxygen is considered to be a potential radiosensitizer as the hypoxic tumor microenvironment is one of the major obstacles of radiotherapy. Administration of oxygen leads to the formation of peroxide in the hypoxic tumor microenvironment resulting in permanent cellular and DNA damage [198]. Additionally, nonmetallic nanomaterials such as carbon nanotubes and selenium nanoparticles also enhance the radiosensitivity of cancer cells via ROS activation and cellular DNA damage [197]. In this context, PL was reported to show similar effects in breast cancer cells. It was found that when PL was used even at a very low concentration of 2.5 μmol/L in combination with X-ray radiation (6 Gy), the level of radiation-induced generation of ROS in cancer cells was improved, and the rate of apoptosis was also enhanced [199]. PL (0-15 μM) was further reported to enhance the radiosensitivity of CRC cells mediated via ROS production, where the radio response of the cancer cells was improved in a concentration-dependent manner. Additionally, in animal models, tumor growth was delayed when PL (2.4 mg/kg) was administered in combination with a single (8 Gy) and fractionated radiations (3 Gy × 3) [129].

RAD001, an inhibitor of mTOR, is an important radiosensitizer used in traditional medical radiotherapy [200]. A study had reported that when oral cancer cells SCC4 and SCC25 were exposed to radiation (0-8 Gy) in combination RAD001 (30 or 300 nM for 1 h), radiosensitivity of the cells increased significantly in 14 days [200]. Interestingly, in a study, oral cancer cell lines SAS and CGHNC8 were subjected to PL, radiation, and a combination of both, and after 5-7 days, it was observed that the radiation sensitivity of the cells treated with the combination was enhanced by 47.5% and 25.63%, respectively [144]. Thus, it is evident that when used in combination with PL, very low intensity of radiation is found to be effective in cancer cells which is extremely important because radiation therapy is associated with numerous side effects such as mutation, alopecia, myelosuppression, etc. Further, PL attenuated the mRNA levels of the cancer stem cells associated markers such as *SOX2*, *NANOG*, and *Oct-4,* ultimately inhibiting cancer stem cell properties which is one of the major obstacles to radiation therapy [144].

Lately, research has been focused on exploring the radiosensitizing efficiency of phytochemicals, such as curcumin, and resveratrol. For instance, the radiosensitizing activity of curcumin and resveratrol was found to be associated with the modulation of the transcription factor, NF-κB, which is known to be involved in the radioresistance of cancer cells [201, 202]. Another study evaluating the radiosensitization activity of curcumin on *in vivo* model of CRC reported that when curcumin (1 g/kg; twice daily) was administered in combination with radiation (4 Gy, twice weekly; given 1 h after curcumin), the tumor size is reduced significantly. In this study, the chemosensitizing potential was also attributed to its ability to inhibit the NF-κB signaling pathway [203]. Similarly, BKM120 (0.25-1 μM) and BEZ235 (0-10 nM) enhance the radiosensitivity of cancer cells by targeting the PI3K-Akt/mTOR pathway [204, 205]. Multiple lines of evidence have suggested that PL treatment significantly modulated the NF-κB and PI3K/Akt/mTOR signaling pathway in different types of cancers, which provides a hint to explore more regarding the radiosensitizing potential of PL.

## Pharmacokinetics and bioavailability of PL

PL is a hydrophobic drug and thereby exhibits very poor solubility in water. Therefore, despite its immense potential as an anti-cancer drug, its low solubility decreases bioavailability and limits its therapeutic efficacy [206, 207]. However, the co-administration of PL with docetaxel enhanced the bioavailability of docetaxel in Sprague-Dawley rats by 1.68-fold, thus acting as a bio enhancer [208]. A study reported that the plasma concentrations of PL, post-administration (50 mg/kg) in rats, were found to be 1511.9 ng/mL, 418.2 ng/mL, and 41.9 ng/mL PL at 30 min, 3 h, and 24 h, respectively [209]. Due to the low bioavailability of PL, the development of novel drug delivery systems is essential for the enhancement of effectiveness *in vivo* [210]. Drug carriers, such as hydrogels, liposomes, microspheres, and nanoparticles, are efficient means of improving the solubility, cellular uptake, and bioavailability of a drug. In addition, these drug delivery systems are also associated with tumor-targeted drug release [207].

Chitosan is a biocompatible natural polymer, and chitosan-based nanoparticles are a safe and efficient drug delivery system. A study showed that PL encapsulated chitosan-based nanoparticles exhibited a high potential for tumor-targeted drug release and showed cytotoxicity against gastric cancer cells by increasing the intracellular ROS. Moreover, it increased the solubility and bioavailability of PL [207]. Another study reported that the encapsulation of PL in chitosan- and fucoidan-based nanoparticles also enhanced its bioavailability and solubility. Furthermore, these nanoparticles induced cytotoxicity against prostate cancer cells by inducing oxidative stress via the excessive formation of ROS [211]. Nanoemulsions are also known to enhance the stability, solubility, and bioavailability of a drug. Pharmacokinetic analysis showed that the orally administered PL-loaded nanoemulsion was rapidly absorbed and slowly eliminated compared to the pure form of the drug. This improved the oral bioavailability of the PL-loaded nanoemulsion by approximately 1.5-fold as compared to the pure form. However, a study showed that the bioavailability of PL following oral administration at 5 mg/kg and 10 mg/kg were 76.39% and 50.08%, respectively [61]. Hence, further studies need to be undertaken in order to elucidate the pharmacokinetic profile of this pleiotropic natural compound.

## Toxicity profile of PL

Analysis of the toxicity profile of a compound is a pre-requisite for developing it as an anti-cancer drug. The non-toxic nature of PL is evidenced by a number of pre-clinical studies. For instance, a study evaluating the effect of paclitaxel and PL nanoformulation on a xenograft model of HCC has evidenced that the combination diminished the toxicity imparted by the native form of the drugs on the adjacent tissues of the tumor [212]. Additionally, the effect of PL on the normal function of the kidney and liver was evaluated by assessing the serum levels of alanine aminotransferase (ALT), urea, aspartate aminotransferase (AST), and creatinine. It was observed that PL reversed the levels of these enzymes, which was shown to be escalated with DMH + DSS treatment, thus showing that PL does not have any adverse effect on the normal functioning of the liver and kidney. Also, on investigating liver sections, no significant alterations were found, thus confirming that PL is not associated with hepatotoxicity [130]. Also, another study showed that though PL exerted significant cytotoxicity on lung cancer cells, it did not hamper the growth of normal lung epithelial cells [138]. Further, toxicological studies also revealed that the oral administration of a PL-nanoemulsion did not exhibit any toxicity in mice for 60 days. However, PL loaded nanoemulsions (10 mg/kg) induced potent anti-tumor activity against the *in vivo* xenograft model of melanoma [206].

## Conclusion and future prospects

PL, the amide alkaloid isolated from the roots and fruits of long pepper, is a potential compound for the prevention and treatment of many different cancers. Since the discovery of its structure in 1968, many researchers have been successful in isolating and synthesizing the compound in laboratories following different approaches from the commercially available precursors. A wide variety of PL derivatives such as L50377 have also been synthesized, mainly exploiting the anti-cancer property exhibiting moiety 3,4,5-trimethoxybenzyl present in PL. PL is also combined with metal complexes such as [RuCl_2_(N-N) (P-P)] and 1,4-bis(diphenylphosphino)butane, PtCl_2_(PPh_3_)_2_, *cis*-[PtCl(PIP-OH)(PPh_3_)_2_]PF_6_ to enhance its therapeutic effects.

PL and its derivatives were reported to exhibit diverse biological activities, including anti-arthritic, anti-asthmatic, anti-diabetic, anti-epileptic, anti-inflammatory, anti-microbial, anti-oxidant, anti-stress, anti-tumor, anti-ulcer, and immunomodulatory activities, which encouraged researchers to explore more about the pharmacological effects of this compound. The anti-cancer property of PL against different types of cancers has been studied in detail in *in vitro* and *in vivo* settings. The ability of PL to modulate the important cell signaling pathways such as PI3K/Akt/ mTOR, NF-κB, JAK/ STAT3, and ERK suggests that this compound is effective in modulating the important hallmarks of cancer, including cell survival, proliferation, invasion, migration, EMT, metastases, and angiogenesis. The multi-targeted and pleiotropic nature of PL suggested that it might also be able to regulate complex phenomena such as chemoresistance and radioresistance, which are the major hindrances towards the current treatment modalities. Further, researchers must focus on exploring the effects of PL in different experimental models for a particular cancer type and in conducting more studies in *ex vivo* and clinical settings.

Recently, the focus was driven towards developing PL as a chemosensitizer and radiosensitizer which sensitized the cancer cells towards the commercially available chemotherapeutics including cisplatin, doxorubicin, 5-FU, gemcitabine, oxaliplatin, and PTX, and ionization/X-ray radiation. PL was found to sensitize the cancer cells to chemotherapeutics and radiation by acting on the cell signaling pathways and genes associated with chemo and radioresistance, inhibiting the biotransformation of the drugs into less active metabolites, and suppressing the properties of cancer stem cells. It was also reported that PL exhibited anti-cancer effects at a very low concentration which tends to reduce the chances of toxicity of the adjacent normal cells, organs, and adverse side-effects. In fact, PL was reported to impart hepatoprotective and cardioprotective effects. Therefore, this compound might be used as an adjuvant in combination with the standard chemotherapeutics to relieve the side effects caused by them to some extent.

The main hindrance towards developing PL as a standard chemotherapeutic is its hydrophobic nature contributing to its low bioavailability. However, studies have evidenced that PL enhanced the bioavailability of docetaxel when used in combination and thus acted as a bio enhancer. Therefore, drug carriers, such as hydrogels, liposomes, microspheres, and nanoparticles formulations can be adapted for enhancing the bioavailability of PL. It was also found that PL-loaded nanoemulsion showed a better pharmacokinetic profile compared to the pure form of this drug.

Although PL has shown immense potential in the prevention and treatment of different types of cancers; however, some studies must be conducted as a pre-requisite to validate the pre-clinical studies and developing it as a clinical chemotherapeutic drug are mentioned below:

- The chemopreventive effects of PL should be evaluated in different experimental models of a particular cancer type.
- Cytotoxicity studies of the compound in different organs should be conducted.
- Molecular markers should be developed to determine the efficacy of PL in randomized multicentered clinical trials.
- The bioavailability of PL and its metabolic and toxicity profile should be studied in detail in humans.
- Effective bioformulations of PL that are designed for sustained release should be developed.

Hence, proper attention should be given to conducting such studies of this compound to develop it as a potential anti-cancer drug. Further, it is worth mentioning that the safe nature of PL strengthens the evaluation of the therapeutic effects of this drug in clinical trials in the future.

## Abbreviations

ABC-DLBCL: activated B cell-like subtype of diffuse large B cell lymphoma
Akt: protein kinase B
Bax: B-cell lymphoma 2 associated X apoptosis regulator
Bcl-2: B-cell lymphoma 2
Bcl-xL: B-cell lymphoma-extra-large
CBR1: carbonyl reductase 1
CCA: cholangiocarcinoma
CDK: cyclin-dependent kinase
CHOP: C/EBP homologous protein
cMET: hepatocyte growth factor receptor
CRC: colorectal cancer
DLBCL: diffuse large B cell lymphoma
DMH: 1,2-dimethylhydrazine
DSS: dextran sulfate sodium
EGFR: epidermal growth factor receptor
ERK: extracellular signal-regulated kinases
FOXO3A: forkhead box O3A
GADD45α: growth arrest and DNA-damage-inducible, alpha
GBM: glioblastoma multiforme
GSH: glutathione
GSTP1: glutathione S-transferase pi 1
HCC: hepatocellular carcinoma
HER: human epidermal growth factor receptor
HK2: hexokinase 2
HNC: head and neck cancer
HO-1: heme oxygenase-1
IL: interleukin
JAK: Janus kinase
JNK: c-Jun N-terminal kinase
LC3: microtubule-associated protein 1 light chain 3
MDA: malondialdehyde
MM: multiple myeloma
MMP-9: matrix metalloproteinases-9
mTOR: mammalian target of rapamycin
NFBTA: (*E*)-*N*-(4-fluorobenzyl)-3-(3,4,5-trimethoxyphenyl) acrylamide
NF-κB: nuclear factor-kappa B
PARP: poly (ADP-ribose) polymerase
PCNA: proliferating cell nuclear antigen
p-eIF2α: phosphorylation of eukaryotic initiation factor-2α
PI3K: phosphoinositide 3-kinases
PL: piperlongumine
PTX: paclitaxel
RCC: renal cell carcinoma
ROS: reactive oxygen species
SOX2: sex determining region Y-box 2
Sp: specificity protein
STAT3: signal transducer and activator of transcription 3
TGF-β: transforming growth factor-beta
TrxR: thioredoxin reductase
VEGF: vascular endothelial growth factor
XIAP: X-linked inhibitor of apoptosis protein
ZEB1: zinc finger E-box binding homeobox 1

## Declarations

### Copyright

© The Author(s) 2021.
